# Joint Formation Control with Obstacle Avoidance of Towfish and Multiple Autonomous Underwater Vehicles Based on Graph Theory and the Null-Space-Based Method

**DOI:** 10.3390/s19112591

**Published:** 2019-06-06

**Authors:** Shi-kun Pang, Ying-hui Li, Hong Yi

**Affiliations:** 1 MOE Key Laboratory of Marine Intelligent Equipment and System, Shanghai Jiao Tong University, Shanghai 200240, China; liyinghui@sjtu.edu.cn (Y.-h.L.); yihong@sjtu.edu.cn (H.Y.); 2 State Key Laboratory of Ocean Engineering, Shanghai Jiao Tong University, Shanghai 200240, China

**Keywords:** formation control, towfish, AUVs, graph theory, obstacle avoidance, NSB

## Abstract

In this study, a new joint formation combined with a two-part underwater towed vehicle (towfish) with multiple autonomous underwater vehicles (AUVs) was investigated. A triangular structure formation was established based on graph theory, in which the main point is the secondary towed vehicle acting as the “leader,” and the other two points are AUVs acting as “followers.” The excellent real-time performance and high flexibility of the towfish is highlighted, and the communication delay and fixed routine of AUVs can be avoided simultaneously. As to the obstacle avoidance, the null-space-based behavioral approach is proposed. On the basis of this approach, the formation task moving to the target is decomposed into different subtasks, and the obstacle avoidance subtask is set as the highest priority. The vector of the low-level task is projected to the null space of the high-level task vector, and the integrated task output is used as the final output function. The low-level task is partially or completely accomplished while handling the higher task; therefore, the mutual conflict between different level targets can be avoided. Moreover, the corresponding task functions are designed in accordance with different subtask priorities. The comprehensive output function of formation motion is deduced and established to ensure that obstacles can be avoided effectively. Furthermore, simulation results demonstrate the effectiveness and feasibility of the proposed method in a complex underwater environment with obstacles.

## 1. Introduction

**Figure 1:**
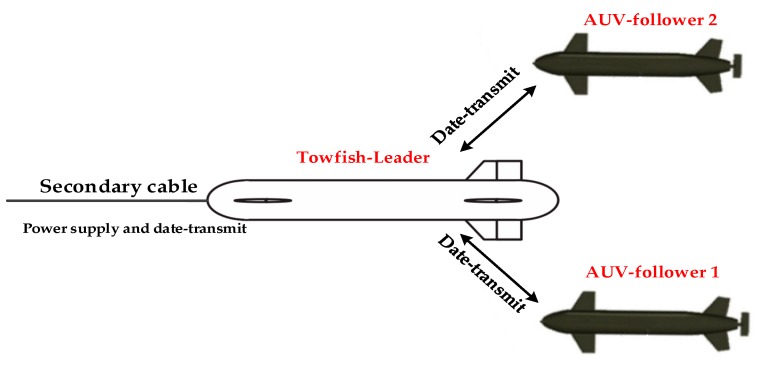
Joint formation of a towfish and two AUVs.

Underwater towed vehicles (towfishes) and autonomous underwater vehicles (AUVs) have been widely utilized in the exploration of underwater environments, including marine magnetic surveying, oceanographic mapping, and geology sampling. A single towfish or AUV can hardly accomplish some complex or high-disk missions, especially in the presence of uncertainties, incomplete information, or distributed control [1,2,3]. For this reason, interest in studying the coordination control of the formation problem of multi-AUVs has increased in recent years. However, an AUV or even an AUV formation has its own disadvantages, such as communication delay, data packet loss, fixed routine, and no real-time communication with the mother ship. However, the towed cable in a towfish system can not only supply continuous power to the towed vehicle but also transmit information between the mother ship and the towed vehicle in real time. If a formation is composed of a towfish and multiple AUVs, then the advantages of a towfish can be strengthened, and the weakness of an AUV can be overcome. In a joint formation, the towfish serves as the “leader” and a relay station, which can receive a minimal amount of detection and sensor data from the AUVs and send the received and its own information to the mother ship on time. In addition, the working routine is decided by the towing mother ship and can be changed as needed. In this manner, the system will be more flexible for practical work. The AUVs act as “followers” and receive instructional messages from the “leader” towfish and transmit the exploration data to the “leader.” SUBNERO M2M series modems, which already have been used in AUV experiments, can be used in the scenario described in this paper. The bandwidth of this device is 16 kHz. This joint formation (shown in Figure 1) can considerably improve working efficiency, save money and time, and expand the scope of the working area. Meanwhile, the obstacle avoidance problem must be considered when the formation is moving toward the target. Hence, this study focuses on the combination of a triangular structure formation and a towfish with two AUVs and the formation obstacle avoidance problem. 

**Figure 2:**
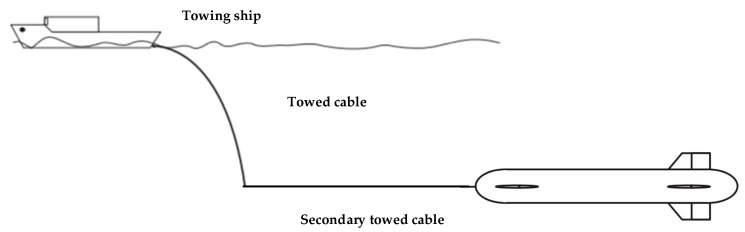
One-part towed vehicle.

**Figure 3:**
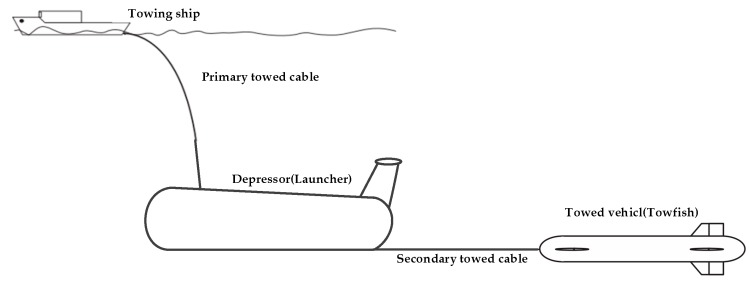
Two-part towed vehicles.

An underwater towed system consists of a towing mother ship, towed cable, and a towed vehicle and can generally be classified into two main types: one-part (shown in Figure 2) [4,5,6,7,8,9] and two-part towed vehicles [10,11,12,13,14,15] (shown in Figure 3). For one-part towed vehicles, the author in [4] proposes a robust motion control method based on a high-gain observer and a linear–quadratic–integral control scheme for a one-part underwater vehicle with movable wings. In [5], the steady-state equilibrium of a towfish is checked and guaranteed to maintain a given depth for monitoring different levels of the column of water. Moreover, a parametric study for the attitude and depth control of the towfish is proposed. In [6], the towed vehicle is simplified as a mass point, and the circular maneuver of the towed cable and vehicle is analyzed. In [7], laboratory experimental observation on the hydrodynamic behavior of a self-stable controllable towed vehicle was reported, which enables us to examine the overall characteristics of a controllable underwater towed vehicle by conducting laboratory tests under different control manipulations and towing conditions. In [8], a newly finite difference method for solving the nonlinear dynamic equations of a towed system was developed. Using this discipline, the nonlinear dynamic equation of an underwater towed system can be solved quickly. An AUV–towfish system was studied in [9], and simulations revealed that the towfish travels a great distance and closely follows the waypoints of the AUV in the vertical direction. As for two-part towed vehicles, hydrodynamic parameters were estimated using Computational Fluid Dynamics (CFD) techniques, and a new convection interpolation scheme called Blended node and cell based upwind scheme (BNCUS) was proposed and numerically tested in [10]. In [11,12], the towfish motion induced by wave-driven disturbances in the vertical and horizontal planes was described using an empirical model of the depressor motion and a spring–damper model of the secondary cable. A nonlinear, Lyapunov-based, adaptive output feedback control law was designed and shown to regulate pitch, yaw, and depth tracking errors to zero. The theoretical and experimental results in the investigation indicate that the hydrodynamic response of a towed vehicle to the wave-induced motion of a towing ship can be significantly reduced by applying a two-part tow method, and a relatively simple control method enables the towed vehicle to travel in a wide range with a stable attitude, which can be seen in [13,14]. In [15], the changing conditions of the motion characteristics of towed vehicles and cable configuration during the turning maneuvers of a towing ship were explored, and the turning radius and depth of the two towed vehicles changed greatly when the mother ship turned at a small radius at a large towing speed, which one should pay special attention to. The above analysis shows that two-part towed vehicles have distinct advantages compared with one-part vehicles. The two-part towed system can effectively reduce the interference of the mother ship motion on the towed body and has a certain heaving compensation function. Furthermore, the secondary towed vehicle is controlled easily at a desired depth or altitude and attitude if it has an active control mechanism similar to propellers and rudders. Hence, we selected the two-part towed vehicles with active control mechanisms as the “leaders” in the formation in this study. 

Traditionally, behavior-based [16,17,18,19], leader–follower [20,21,22], graph theory [23,24,25,26], virtual structure [27,28,29], and artificial potential methods [30,31,32] are available for the cooperative motion control problem of multiple AUVs. A previous study [16] proposed a new algorithm for controlling a formation of multiple autonomous aerial vehicles based on the null space method. In [17], five sub-behaviors were designed for the formation control mission via the behavior-based formation control system. In [18], a drone formation based on self-organizing behavior was designed for Unmanned Aerial Vehicles (UAVs), and the results verified the feasibility and effectiveness of the algorithm. In [19], the application of the null-space-based (NSB) method to a fleet of marine surface vessels was presented, and the method was considered as a guidance system to perform complex missions in realistic scenarios. A linear PID controller was used to control each single quadrotor, and a slide mode controller, which uses the leader–follower structure, was adopted to solve the formation flying problem in [20]. In [21], an event-based leader–follower strategy for the synchronization of multiagent systems was considered. In [22], three types of AUV formation were introduced on the basis of the leader–follower strategy. In [23], a multiquadrotor formation control scheme based on graph theory was proposed for controlling three quadrotors performing a triangular structure. In [24], we concluded that using graph theory is an efficient way to realize AUV formation under uncertain communication conditions. In [25], multibody dynamics, relative coordinates, and graph theory were combined to analyze the structure of a vehicle suspension and provide a new solution for vehicle dynamics modeling. In [26], the graph theory was used for the path planning of AUV docking in a stationary obstacle environment. In [27], a virtual structure combined with an artificial potential field algorithm was proposed to solve the obstacle avoidance problem on the AUV formation proceeding. In [28], a distributed dynamic controller was adopted for the formation keeping of underactuated vehicles with exponential convergence to guarantee the formation error to zero using the virtual approach. In [29], the paths ready for coordinate AUVs were flexible, using the methods of formation reference point and virtual structure. In [30], the gravitational function of the artificial potential field method was adopted to obtain a sufficient smooth flight path for a UAV. In [31], an improved artificial potential field method was proposed to solve the problem of UAV formation control with obstacle avoidance in a complex environment. In [32], a novel distributed formation control strategy was proposed on the basis of the integration of a radial basis function neural network with the artificial potential field method. The wireless sensors problem is addressed in [33,34]. The Improved Adaptive Probabilistic Search algorithm proposed in [33] is fully distributed and bandwidth efficient in peer-to-peer networks. And an effective technique is adopted in [34] for preserving k-coverage and the reliability of data with logical fault tolerance, which attempts to select an efficient route for transferring the information.

Under comprehensive and comparative consideration, the joint formation control of a towfish and multiple AUVs is based on the graph theory in this study, because the mature theory and mathematical tools of this strategy can easily handle related issues in formation control and can be more flexible when combined with other methods of managing the obstacle avoidance problem. 

For the joint formation obstacle avoidance issue, the behavior-based NSB method [35] is adopted. On the basis of the behavioral method, the task objective is decomposed into a series of basic sub-behaviors, such as perception, detection, obstacle avoidance, and planning, using a bottom-up system construction process. Then, a parallel control loop is formed, and the corresponding target task can be accomplished by acting on certain actuators through coordination and cooperation. The behavior-based strategy has the advantages of a quick response to the environment and ability to be flexibly extended to the entire system, because a single behavior requires only a simple task to be completed. Compared with other behavior-based obstacle avoidance methods [36,37,38,39], the NSB method has other characteristics [40,41,42]. It can fully utilize the zero space of a high-priority task to complete low hierarchical tasks while maintaining the primary task until it is completely finished. Furthermore, it also has a strong real-time performance and no conflicts between subtasks. 

As for the deep tow system shown in Figure 3, the two-part towing arrangement, including the surface mother towing ship, primary towed cable, launcher (depressor or primary towed vehicle), secondary towed cable, and the secondary towed vehicle (towfish), is adopted. The launcher stores the towfish in the hangar before or after it is released or restored. The launcher equipped with sensors, such as a depthometer, underwater camera, and side scan sonar, is deployed from the mother ship before the joint formation starts to work. After ensuring that no suspended items, such as marine cables or big obstacles in the working area, are present, the towfish is released from the launcher. The neutrally buoyancy of the controllable towfish ensures a high performance in depth or altitude and attitude control, but for security reasons, the towfish is usually designed to have some positive buoyancy. In case the towed cable is broken or something unexpected happens, the towfish can float to the sea surface. When the towfish reaches the set depth or altitude, and the motion state is stable, AUV followers are then deployed to the neighborhood of the towfish. Using the range and bearing sensors, the AUV can measure the line-of-sight distance between the centers of the follower AUVs and leader towfish and the angles with respect to the target. The formation controller enables AUVs to track the towfish, and the designed formation structure can be established throughout the whole working period. The contents of this study can be summarized as follows. Any two AUVs and a lead towfish can theoretically form a triangle, and in order to simplify the simulation scenarios, a special isosceles triangle is chosen as the research object in this paper. First, the triangular structure joint formation of a towfish and two AUVs is established based on graph theory. With the secondary controllable towed vehicle of the two-part towed system as the main point in the formation structure, the motion state of the towfish can be adjusted easily and has less interference from the sea surface and the towing mother ship. The two AUVs can communicate with the towfish online. The joint formation can work more efficiently with flexible routes and low cost. Second, the NSB approach combined with the graph theory is adopted to solve the formation obstacle avoidance problem while approaching the target to ensure object integrity. Finally, the forming and obstacle avoidance ability of the joint formation are verified through simulation. 

The rest of this study is organized as follows: In the next section, the mathematical model of the towfish and AUV is established. Section 3 shows the practical formation controller with obstacle avoidance of the system. Section 4 provides the numerical results to evaluate the formation controller performance. Finally, conclusions are drawn in Section 5.

## 2. Mathematical Model of Towfish and AUV

The towfish and AUV used in this study are derived from [43,44]. The AUV in the references can be treated as a controllable towfish when it is connected by the towed cable to the launcher or mother ship. Three degrees of freedom (DOF) are available in surge, sway, and yaw, because the formation is moving in a 2D plane. When the speed of the mother ship is low (no more than 1 kn), the state of the towfish works steadily, such as an AUV with a cable. When the obstacle is imminent, the towfish can actively avoid the obstacle on the basis of the designed controller according to sensing information. Related references [45,46] state that in scenarios in which the towfish is moving in a small area, the influence of the towed cable can be neglected, and the towfish is regarded as an AUV. The model adopted in this paper can be considered as streamlined and symmetrical. On the basis of the above analysis, the 3-DOF kinematics and dynamics model [47,48] of the towfish and AUV without external disturbances is described as follows:

(1) $$\dot{\eta }=J(\eta )v$$

(2) $$M\dot{v}=-C(v)v-\mathrm{Dv}+\tau ,$$

where $J=\left[\begin{array}{ccc} \cos\psi & -\sin\psi & 0\\ \sin\psi & \cos\psi & 0\\ 0 & 0 & 1 \end{array}\right]$, $M=\left[\begin{array}{ccc} m_{11} & 0 & 0\\ 0 & m_{22} & 0\\ 0 & 0 & m_{33} \end{array}\right]$, $C=\left[\begin{array}{ccc} 0 & 0 & -m_{22}v\\ 0 & 0 & m_{11}u\\ m_{22}v & -m_{11}u & 0 \end{array}\right]$, $D=\left[\begin{array}{ccc} d_{11} & 0 & 0\\ 0 & d_{22} & 0\\ 0 & 0 & d_{33} \end{array}\right]$, and $\eta ={\left[x\;y\;\psi \right]}^{T}$ denotes the position and heading direction in the earth-fixed frame; $v={\left[u\;v\;r\right]}^{T}$ is the velocity in the body-fixed frame (μ: surge, v: sway, r: yaw); and $\tau ={\left[\tau_{\mu },0,\tau_{r}\right]}^{T}$ is the control signal ($\tau_{\mu }$: surge force, $\tau_{r}$: yaw moment). The Appendix A is the table of some representative symbols and meaning of them in this paper. The model in this paper has two horizontal propellers and can adjust the yaw moment depending on different revolving speeds and directions. The following assumptions are drawn because of the limited velocities and input forces in consideration of actual situations and to facilitate subsequent controller design and simulation.

***Assumption 1***: The velocities and input forces are bounded as $\sup_{t}\left\|u\right\|={\bar{u}}_{\max}$, $\sup_{t}\left\|v\right\|={\bar{v}}_{\max}$, $\sup_{t}\left\|r\right\|={\bar{r}}_{\max}$, $\sup_{t}\left\|\tau_{u}\right\|={\bar{\tau }}_{u\max}$, and $\sup_{t}\left\|\tau_{r}\right\|={\bar{\tau }}_{r\max}$ with known ${\bar{u}}_{\max}>0$, ${\bar{v}}_{\max}>0$, ${\bar{r}}_{\max}>0$, ${\bar{\tau }}_{u\max}>0$, and ${\bar{\tau }}_{r\max}>0$.
***Assumption 2:*** The vehicle can timely transmit a minimal amount of information, including orientation, velocity, and certain sensor data between each other in a short distance (the largest distance is no more than 6 m in this study).
***Assumption 3:*** Each vehicle can obtain its own orientation and velocity information accurately and in real time from the onboard sensors. No disturbances and obstacles are considered in this formation system.

## 3. Controller Design

In this article, the joint formation that combines a towfish with two AUVs is established based on graph theory. In the triangular structure formation, the towfish is the main point, and the other two AUVs act as followers on the rest of the points. For easy description, the agent towfish and AUVs are numbered as *i* (*i* = 1 for towfish, *i* = 2 for the left AUV, and *i* = 3 for the right AUV). 

### 3.1. Formation Control Based on Graph Theory

Each formation agent consists of a scalar $x_{i}\in R$, which will be coordinated with the rest of the group and can communicate with other agents. This is modeled by a directed graph G. Furthermore, formation mission velocities are created by agent 1 and distributed to other agents under the assumption that the other two agents (AUVs) are connected to the leader. In addition, this kind of connection is not connected using physical cables, but AUVs are “connected” to the towfish by signals.

The first objective is to develop the feedback laws [49] that guarantee the following behavior:

(1) Each agent achieves in the limit a velocity vector $v(t)\in R^{p}$ prescribed for the group; that is,

(3) $$\underset{t\to \infty }{\lim}\left|\dot{x}-v(t)\right|=0,\;i=1,\ldots ,N.\;$$

The vector here is a column vector and the set of p by 1 real vectors are donated by ${\mathbb{R}}^{{}^{p}}$.

(2) If agents i and j are connected by link k, then the difference variable $z_{k}$

(4) $$z_{k}:=\sum_{l=1}^{\ell }d_{lk}x_{l}=\left\{ \begin{array}{l} x_{i}-x_{j}\;if\;k\in L_{i}^{+}\\ x_{j}-x_{i}\;if\;k\in L_{i}^{-} \end{array}\right.$$

converges to a prescribed compact set $A_{k}\subset {\mathbb{R}}^{p}$, k=1,⋯ℓ. The set of links for which the node is positive is set as $L_{i}^{+}$ and for negative is $L_{i}^{-}$. Denoted by ℓ, the total number of links, the N×ℓ incidence matrix D of an undirected graph G is defined as

(5) $$d_{ik}=\left\{ \begin{array}{ll} +1 & if\;k\in L_{i}^{+}\\ -1 & if\;k\in L_{i}^{-}\\ 0 & otherwise \end{array}\right..$$

The target set $A_{k}$ will have different forms depending on the application. Moreover, in this research, it is designed to set the agents to a specified distance. 

Some concatenated vectors are introduced to ensure that the target set $A_{k}$ is feasible [49]:

(6) $$x:={\left[x_{1}^{T},\cdots ,x_{N}^{T}\right]}^{T},$$

(7) $$z:={\left[z_{1}^{T},\cdots ,z_{\ell }^{T}\right]}^{T}.$$

D can be partitioned in terms of its column vectors:

(8) $$D=\left[D_{1}|\cdots |D_{\ell }\right].$$

Moreover, Equation (4) can be written as

(9) $$z_{k}=(D_{k}^{T}⊗I_{p})x.$$

Concatenating $z_{k}$’s together, we obtain:

(10) $$z=(D_{}^{T}⊗I_{p})x$$

which means that z is restricted in the range space $R(D_{}^{T}⊗I_{p})$. Thus, for objective A2 [49] to be feasible, target set $A_{k}$ must satisfy

(11) $$x\{A_{1}\times \cdots A_{\ell }\}\cap R(D_{}^{T}⊗I_{p})\neq \emptyset$$

#### 3.1.1. Passivity-Based Design Procedure

Step 1. Internal feedback

Suppose that the input–output dynamics of agent i are given by

(12) $$x_{i}=H_{i}{}^{o}\{t_{i}\}.$$

**Figure 4:**
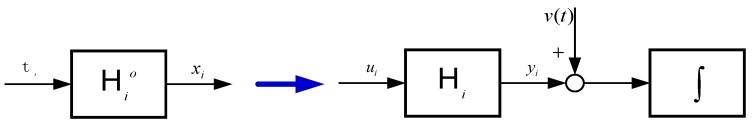
Description of Step 1. Step 1 transforms agent dynamics from (12) to (13) by designing an internal feedback $t_{i}$. The resulting passive block is denoted by $H_{i}{}^{o}$.

The system $H_{i}{}^{o}$ (in Figure 4) describes the input–output dynamics with $t_{i}$ as the input vector and $x_{i}$ as the output. The goal is to make the system passive from an external feedback signal $u_{i}$ (to be designed in Step 2) to the velocity vector:

(13) $$y_{i}:={\dot{x}}_{i}-v(t),$$

where v(t) is a leader towfish velocity of the formation. Moreover, an internal feedback controller $t_{i}$ is created. The feedback system is often described by a transfer function and checked for positive realness, which proves to be passive. For more detail and definition of passivity, please see Appendix B.

Then, the system (AUV models) can be expressed as follows:

(14) $${\dot{x}}_{i}(t)=H_{i}\{u_{i}(t)\}+v(t).$$

and $H_{i}$ is designed to be strictly passive.

$H_{i}$ can either be a dynamic or static nonlinear system. If $H_{i}$ is dynamic, then it is assumed to be in the form

(15) $$H_{i}:=\left\{ \begin{array}{l} {\dot{\xi }}_{i}=f_{i}(\xi_{i},u_{i})\\ y_{i}=h_{i}(\xi_{i},u_{i}) \end{array}\right.,$$

where $y_{i}$ is the velocity error, and $\xi_{i}\in {\mathbb{R}}^{ni}$ is the state variable of the subsystem $H_{i}$. Assume that $f_{i}(\cdot ,\cdot )$ and $h_{i}(\cdot ,\cdot )$ are $C^{2}$ functions, such that

(16) $$f_{i}(0,u_{i})=0\Rightarrow u_{i}=0,$$

(17) $$h_{i}(0,0)=0.$$

Hence, $H_{i}$ is strictly passive with $C^{1}$, positively definite, and has a radially unbounded storage function, making the origin of $H_{i}$ globally asymptotically stable.

This study involves a static system and is restricted to be of the form

(18) $$y_{i}=h_{i}(u_{i}),$$

where $h_{i}:{\mathbb{R}}^{p}\to {\mathbb{R}}^{p}$ is a locally Lipschitz function satisfying

(19) $$y_{i}=u_{i}{}^{T}h(u)>0\;\forall u_{i}\neq 0,$$

thus making it strictly passive.

For the AUV model described in Section 3.1, the mathematical model can be expressed as

(20) $${\dot{x}}_{i}={\dot{\eta }}_{i}=t_{i}={J(\eta )v}_{i}.$$

Define the internal feedback as

(21) $$t_{i}=u_{i}+v(t),$$

then the following transformed system is

(22) $$H_{i}=h(u_{i})=u_{i}.$$

$y_{i}=h(u_{i})=u$ can satisfy Equation (19), because $u_{i}{}^{T}u_{i}>0\;\forall u_{i}\neq 0$, rendering the transformed system strictly passive from $u_{i}$ to $y_{i}$.

Step 2. External feedback

**Figure 5:**
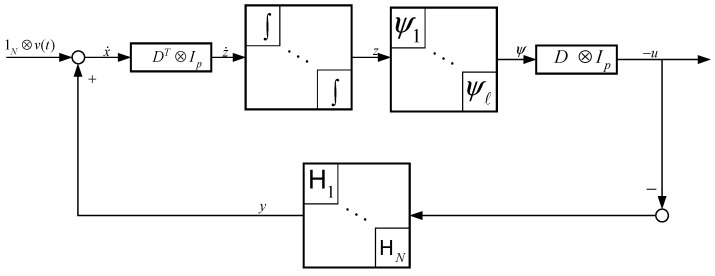
A feedback block diagram of the interconnected multirobot system with feedback.

The setup given in Step 1 is prepared before the suggested control law is presented. Agent dynamics i∈{1,⋯,N} can be concatenated to form a single block diagram. By post multiplying x with $D_{}^{T}⊗I_{p}$ and combining with Equation (10), we obtain the structure in Figure 5, where

(23) $$y:={\left[y_{1}^{T},\cdots ,y_{N}^{T}\right]}^{T}\in {\mathbb{R}}^{pN},$$

(24) $$u:={\left[u_{1}^{T},\cdots ,u_{N}^{T}\right]}^{T}\in {\mathbb{R}}^{pN}.$$

Using a feedback term of the form

(25) $$u_{i}=-\sum_{k=1}^{\ell }d_{ik}\psi (z_{k}),$$

where $z_{k}$ are the difference variables introduced in Equation (3), and $\psi (z_{k}):{\mathbb{R}}^{p}\to {\mathbb{R}}^{p}$ are nonlinearities to be designed. Intuitively speaking, the feedback term applies an input $u_{i}$ as a sum of some function ψ on the difference variables $z_{k}$ for each link.

The $u_{i}$ in (25) can be expressed as

(26) $$u_{i}=-\left[d_{i1}I_{p}\right]\cdots \left[d_{i\ell }I_{p}\right]\psi ,$$

and $\psi :={\left[\psi_{1}^{T},\cdots ,\psi_{\ell }^{T}\right]}^{T}\in {\mathbb{R}}^{p\ell }$ $\psi :={\left[\psi_{1}^{T},\cdots ,\psi_{\ell }^{T}\right]}^{T}\in {\mathbb{R}}^{p\ell }$.

Hence, the concatenation of $u_{i}$ for each i in (26) can be written as

(27) $$u=-(D⊗I_{p}).$$

Furthermore, $\dot{z}$ in Figure 5 and $\dot{z}=(D^{T}⊗I_{p})$ are implied in (10).

#### 3.1.2. Design Criteria for the Feedback

This feedback structure aims to render the entire system in Figure 5 passive. The feedback path from u to y is passive by design, and $\psi_{k}$ must be designed such that the system is passive from $\dot{z}$ to ψ. The nonlinearity form is designed to accomplish

(28) $$\psi_{k}(z_{k})=\nabla P_{k}(z_{k}),$$

where $P_{k}(z_{k})$ is a non-negative C^2^ function. For the formation problem discussed in this study, $P_{k}$ is set to have the following property:

(29) $$z_{k}^{T}\nabla P_{k}(z_{k})=z_{k}^{T}\psi_{k}(z_{k}),\;\forall z_{k}\neq 0.$$

In this manner, $\psi_{k}$ is a monotone function that belongs to the sector [0,∞], making it passive from $z_{k}$ to $\psi_{k}(z_{k})$.

### 3.2. NSB Method for Formation Obstacle Avoidance 

#### 3.2.1. Introduction to NSB

The NSB method first decomposes the overall task into several individual control subtasks and then establishes a task function for each subtask to ensure that each function can complete the corresponding control target. Then, the subtasks are divided into different priorities. The low priority task vector is projected to the null space of the high-priority task vector. Finally, the overall integrated output function of the task is obtained and passed to the underlying actuator to control the vehicle motion in the formation. For the vehicle adopted in this study, σ is the control variable of the control target, and the function model is

(30) σ=f(η).

Its derivative is

(31) $$\dot{\sigma }=\frac{\partial f(\eta )}{\partial \eta }v=J(\eta )v,\;$$

where J(η) is a configuration-dependent task Jacobian matrix. Solving Equation (31) with respect to the minimum norm velocity using least squares,

(32) $$v_{d}=J^{†}({\dot{\sigma }}_{d}+\Lambda \tilde{\sigma }),$$

where $J^{†}$ is the pseudoinverse $J^{†}=J^{T}{\left({\mathrm{JJ}}^{T}\right)}^{-1}(J\neq 0)$, ${\tilde{\sigma }=\sigma }_{d}-\sigma$, Λ>0 is the gain matrix, and $\sigma_{d}$ is the desired value for a certain task. 

Use i to indicate the task priority, and the priority is highest when i=1. Meanwhile, the velocity output of the ith i’th priority task is

(33) $$v_{i}=J_{i}^{†}({\dot{\sigma }}_{i,d}+{\Lambda \tilde{\sigma }}_{i}).$$

The null space projection from the ith i’th priority task to the higher priority is

(34) $$N_{i}(J)=I-J_{i}^{†}J_{i}.$$

Projecting the low-priority task into the high-priority task gives the desired velocity $v_{d}$, and for an *n* task system, the desired velocity becomes

(35) $$v_{d}=v_{1}+\sum_{i=2}^{n}N_{i}(J)v_{i}.\;$$

For *n* = 3, the desired velocity $v_{d}$ is

(36) $$v_{d}=v_{1}+N_{1}(v_{2}+N_{2}v_{3}).$$

#### 3.2.2. Tasks for Obstacle Avoidance

Using the NSB control method for obstacle avoidance, the controller must reach a target while avoiding obstacles. The total task must be decomposed into some subtasks (“Obstacle avoidance” and “Go to target”) to be accomplished. 

Task 1: Obstacle avoidance 

In a multitask control mission, the obstacle avoidance task is set as the highest priority to ensure the integrity of the agent. The distance from the vehicle to the obstacle center is set as the task variable $\sigma_{1}$, and $\sigma_{1,d}$ is the safety distance d, at which the vehicle should stay outside of

(37) $$\sigma_{1}=\left\|\eta -P_{0}\right\|$$

(38) $$\sigma_{1,d}=d,$$

where $P_{0}$ is the position of the obstacle center. Moreover, the corresponding Jacobi matrix is

(39) $$J_{1}=\frac{\eta -P_{0}}{\left\|\eta -P_{0}\right\|}={\hat{r}}^{T},$$

where ${\hat{r}}^{T}$ is the unit vector aligned with the vehicle-to-obstacle direction, and ${\hat{r}}^{T}$ exists if and only if

(40) $$\eta \neq P_{0}.$$

The corresponding desired velocity for Task 1 is

(41) $$v_{1}=J_{1}^{†}\Lambda_{1}(d-\left\|\eta -P_{0}\right\|).$$

Moreover, the null space $N_{1}$ is

(42) $$N_{1}=I-J_{1}^{†}J_{1}=I-{\hat{r}\hat{r}}^{T}.$$

Task 2: Go to target

This task enables the formation to go to the target using the shortest path after the obstacle is avoided. The task variables are

(43) $$\sigma_{2}=\eta ,$$

(44) $$\sigma_{2,d}=P_{t\arg et}.$$

Analogies, such as task 1, the Jacobi matrix, velocity output, and null space are

(45) $$J_{2}=I,$$

(46) $$v_{2}=\Lambda_{2}(p_{t\arg et}-\eta ),$$

(47) $$N_{2}=0.$$

According to Equation (35), the desired velocity vectors become

(48) $$v_{d}=v_{1}+N_{1}v_{2}.$$

### 3.3. Formation Controller Design with Obstacle Avoidance Function

#### 3.3.1. Control Scheme Based on NSB Strategy

**Figure 6:**
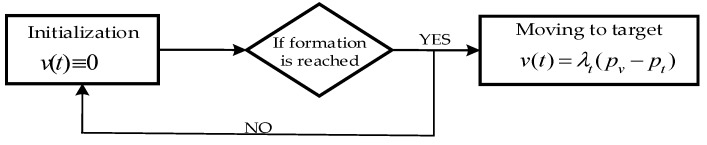
Two parts before or after obstacle avoidance.

**Figure 7:**
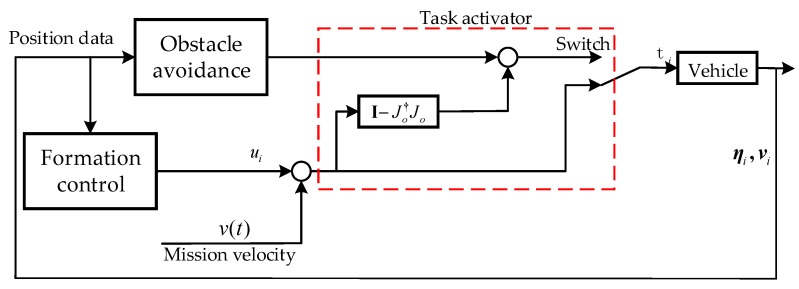
Control scheme for formation with obstacle avoidance function.

Before or after avoidance, the simulation consists of two separate states shown in Figure 6. The first state is initialization, in which the vehicles reach the desired formation. In this state, the formation mission velocity is v(t)=0. The second state starts after the formation is reached, which is moving to the target. At this time, the mission velocity is activated in Figure 7. 

The NSB control scheme introduced earlier can provide a quite useful framework for cooperative tasks, because the output u(i) from the formation can be considered as a separate task. Keeping the integrity of vehicles is important, and the mission is decomposed into two tasks, that is, obstacle avoidance of the highest priority and going to the target of the lowest priority. Thus, the control logical scheme based on NSB strategy is shown in Figure 7.

#### 3.3.2. Stability Analysis 

The stabilities of the formation controller based on graph theory and NSB-based obstacle avoidance is analyzed, respectively, to investigate the stability of the total system. 

• Stability of the formation controller

From Figure 5, the set of equilibria is given by

(49) $$\Omega =\{(z,\xi )|\xi =0,(D⊗I_{p})\psi (z)=0\;and\;z\in R(D_{}^{T}⊗I_{p})\}.$$

The equilibrium points of the interconnected system must be inside the target set $A_{k}$. As observed from Equations (15)–(17), and combined with the strictly passive property, the equilibrium ξ=0 only occurs when u=0, making $(D⊗I_{p})\psi (z)=0$. This result is satifactory, because ξ=0⇒y=0 is the goal defined in Equation (3). As observed from (29) and the agreement problem (where the goal for the agents is to agree on a value, i.e., $z_{k}=0$), if and only if z=0 z=0, z and ψ(z) are orthogonal to each other due to the fact that $z\in R(D_{}^{T}⊗I_{p})$ and $\psi (z)\in N(D⊗I_{p})$. In this manner, the set of equilibrium points of $(D⊗I_{p})\psi (z)=-u=0$. For more passivity and asymptotic stability of the agreement problem, refer to [47]. Then, the stability of the graph theory-based formation problem is improved. 

• Stability analysis of the NSB method

One must evaluate the convergence of each task variable separately to analyze the convergence of the global task to verify if the NSB can complete all tasks. Start with Equation (48), multiply with $J_{1}$, and observe that $J_{1}(I-J_{1}^{†}J)=0$,

(50) $$J_{1}v_{d}=J_{1}v_{1}.$$

Using Equations (32) and (33) for i=1 and inserting for $v_{d}$ and $v_{1}$, the error dynamic of the first task is

(51) $${\dot{\sigma }}_{1}={\dot{\sigma }}_{1,d}{+\Lambda }_{1}(\sigma_{1,d}-\sigma_{1}),$$

(52) $${\dot{\tilde{\sigma }}}_{1}{=-\Lambda }_{1}{\tilde{\sigma }}_{1},$$

where $\tilde{\sigma }=\sigma_{1,d}-\sigma_{1}$ and ${\dot{\tilde{\sigma }}=\dot{\sigma }}_{1,d}-{\dot{\sigma }}_{1}$. By using the Lyapunov function and Theorem 4.1 in [50], the proof that $\sigma_{1}$ converges toward $\sigma_{1,d}$ is straightforward:

(53) $$V_{1}=\frac{1}{2}{\tilde{\sigma }}_{1}P{\tilde{\sigma }}_{1}{}^{T},$$

where $V_{1}(0)=0,\;V_{1}({\tilde{\sigma }}_{1})>0\;in\;\partial -[0]$, ${\dot{V}}_{1}={\dot{\tilde{\sigma }}}_{1}P{\tilde{\sigma }}_{1}{}^{T}$, and ${\dot{V}}_{1}=-\Lambda_{1}{\tilde{\sigma }}_{1}P{\tilde{\sigma }}_{1}{}^{T}\;{\dot{V}}_{1}<0\;in\;\partial -[0]$. ∂ is the domain containing ${\tilde{\sigma }}_{1}=0$, and Λ and P are positively definite; thus, ${\tilde{\sigma }}_{1}=0$ is asymptomatically stable, which means that ${\tilde{\sigma }}_{1}$ converges to zero and $\sigma_{1}$ converges toward $\sigma_{1,d}$. In this manner, the primary task is always fulfilled. 

The second task is only fulfilled if the higher primary task has no complications. By multiplying Equation (48) with $J_{2}$ and noting that $N_{2}=0$,

(54) $$v_{d}=J_{2}v_{1}+J_{2}(I-J_{1}^{†}J_{1})v_{2}.$$

Using Equations (32) and (33) for i=1,2 and inserting for $v_{d}$, $v_{1}$, and $v_{2}$, we obtain

(55) $${\dot{\sigma }}_{2}{=J}_{2}J_{1}^{†}({\dot{\sigma }}_{1,d}+\Lambda_{1}{\tilde{\sigma }}_{1}){\dot{\sigma }}_{1,d}{+J}_{1}(I-J_{1}^{†}J_{1})J_{2}^{†}({\dot{\sigma }}_{2,d}+\Lambda_{2}{\tilde{\sigma }}_{2}).$$

Assuming no complications,

(56) $$J_{2}J_{1}^{†}=0.$$

Then, the dynamic error for Task 2 is

(57) $${\dot{\tilde{\sigma }}}_{2}{=-\Lambda }_{2}(\sigma_{2,d}-\sigma_{2}).$$

Similar to Equation (53),

(58) $$V_{2}=\frac{1}{2}{\tilde{\sigma }}_{2}P{\tilde{\sigma }}_{2}{}^{T},$$

where $V_{2}(0)=0,\;V_{2}({\tilde{\sigma }}_{2})>0\;in\;\partial -[0]$, ${\dot{V}}_{2}={\dot{\tilde{\sigma }}}_{2}P{\tilde{\sigma }}_{2}{}^{T}$, and ${\dot{V}}_{2}=-\Lambda_{2}{\tilde{\sigma }}_{2}P{\tilde{\sigma }}_{2}{}^{T}in\;\partial -[0]$, which is negatively definite, and ${\tilde{\sigma }}_{2}$ is asymptotically stable and will converge to zero (i.e., $\sigma_{2}$ converges toward $\sigma_{2,d}$). The assumption that $J_{2}J_{1}^{†}=0$ can verify that the point $\sigma_{2,d}$ is on the stable circle; thus, the NSB controller can be used for multiple tasks. 

As mentioned previously, the stabilities of formation control and the NSB method have been proven, which will be a brief qualitative proof that such an interconnection system is stable. Assuming that the desired formation $X^{f}$ does not violate the minimum distance $d_{o}$ in the obstacle avoidance control, then it is easily seen that the same structure as earlier is retained when the obstacle avoidance control is deactivated. When the obstacle collision task is activated, the NSB framework can guarantee the stability of each task as long as they do not violate each other. Based on the initial assumption, this scenario makes the interconnected controller system stable.

## 4. Simulation and Analysis

Several computer simulation conditions are studied in this section to validate the effectiveness of the proposed formation controller with the obstacle avoidance function. For the following cases, the maximum velocity for the vehicle is 1 m/s. 

• Case 1

**Figure 8:**
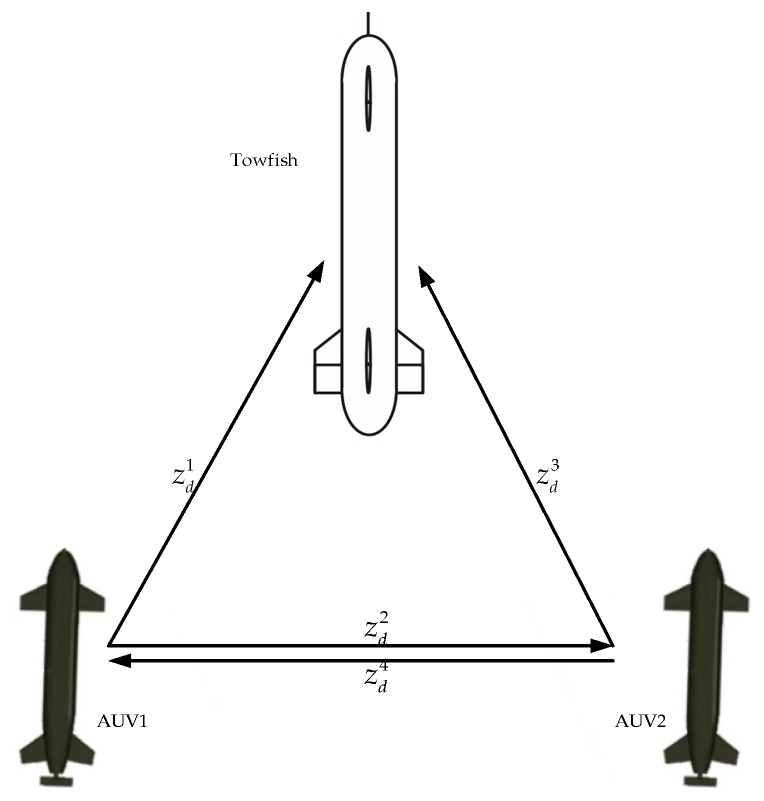
Formation structure.

**Figure 9:**
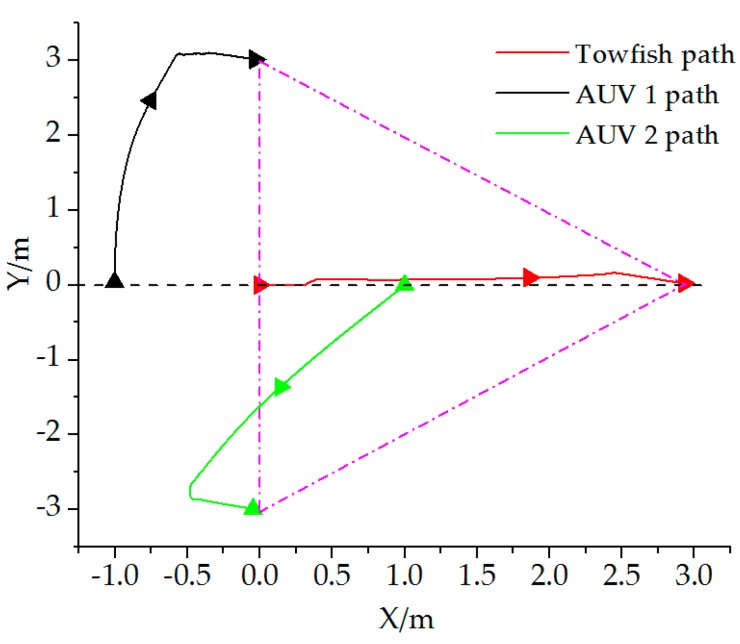
Vehicle paths while forming formation.

**Figure 10:**
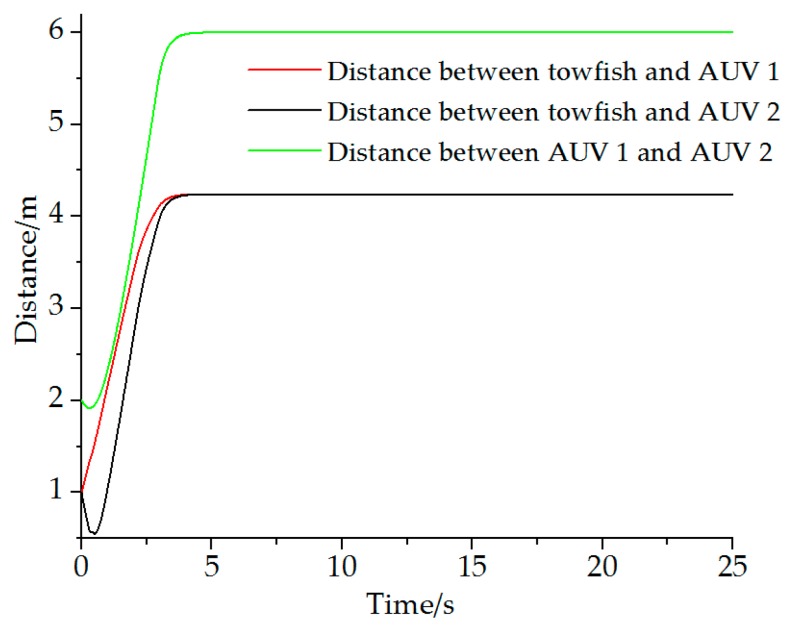
Distance between vehicles.

First, the formation control based on graph theory is tested. In this case, we focus our attention on the formation, and the yaw angle of the vehicle is neglected. The initial positions of the vehicles are set as:

Leader towfish: $x_{1}=\eta_{towfish}={\left[0\;0\;-\right]}^{T}$, AUV 1: $x_{2}=\eta_{AVU1}={\left[-1\;0\;-\right]}^{T}$, and AUV 2: $x_{3}=\eta_{AVU2}={\left[1\;0\;-\right]}^{T}$. The desired distance-based formation structure (Figure 8) is $X^{f}={\left[\begin{array}{l} \begin{array}{cc} 3 & 0 \end{array}\\ \begin{array}{cc} 0 & 3 \end{array}\\ \begin{array}{cc} 0 & -3 \end{array} \end{array}\right]}^{T}$. The incidence matrix D for this test is $D=\left[\begin{array}{cccc} 1 & 0 & 1 & 0\\ -1 & 1 & 0 & -1\\ 0 & -1 & -1 & 1 \end{array}\right]$. The simulation time is 25 s, and the mission velocity is v(t)=0. Results are shown in Figure 9 and Figure 10. 

Results show that the desired formation can be formed on the basis of the proposed theory. The towfish and AUVs move to the set positions during the first 5.5 s. When the joint formation is formed, the vehicles stay at the locations at a fixed distance as desired. 

• Case 2

**Figure 11:**
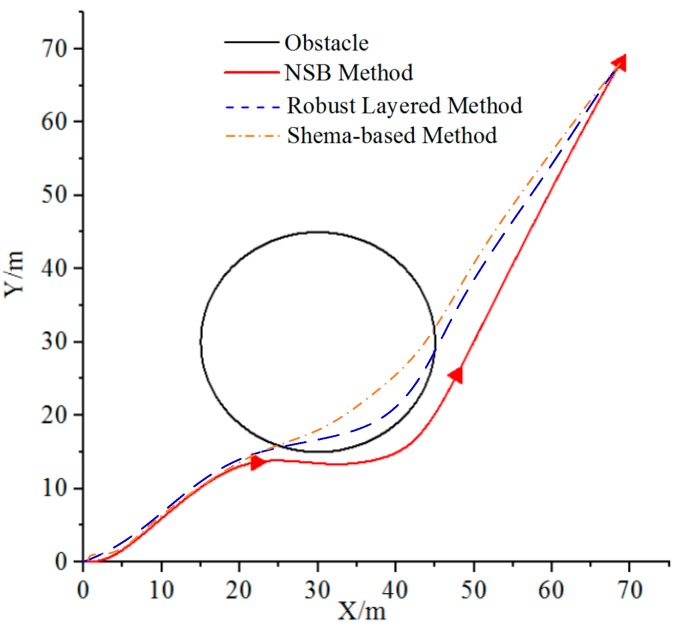
Obstacle avoidance of different methods.

Before the NSB method is used for obstacle avoidance, some other behavior-based theories are studied together to compare the avoidance effect, and results are shown in Figure 11. In the simulation, the initial position of an AUV is $\eta_{start}={\left[0,\;0,\;0\right]}^{T}$, and the target is $\eta_{t\arg et}={\left[70,\;70,\;-\right]}^{T}$. The information of the obstacle is $Obstacle={\left[30,\;30,\;15\right]}^{T}$, where the first columns are the position of the obstacle, and the last column is the safety radius $d_{o}$. Figure 11 shows that the NSB method has evident advantages compared with other robust layered and schema-based methods. The NSB method can project the low-priority vector to the null space of the high-priority task according to the design scheme, handle conflicts between different priority tasks, and finally complete the task satisfactorily. On the basis of the above analysis, the joint formation obstacle avoidance simulation is performed using the NSB method.

**Figure 12:**
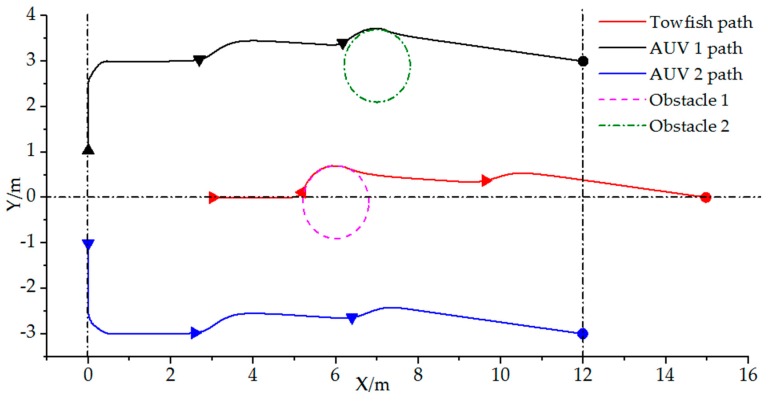
Vehicle formation paths with obstacle avoidance.

**Figure 13:**
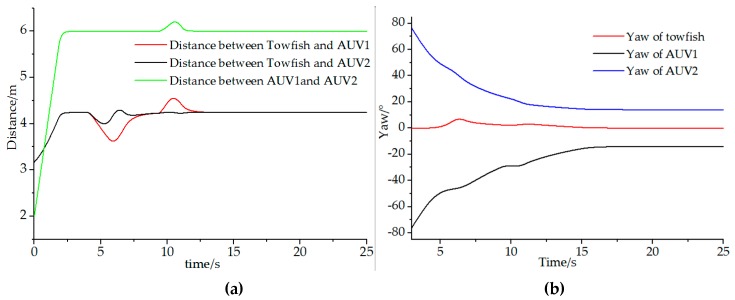

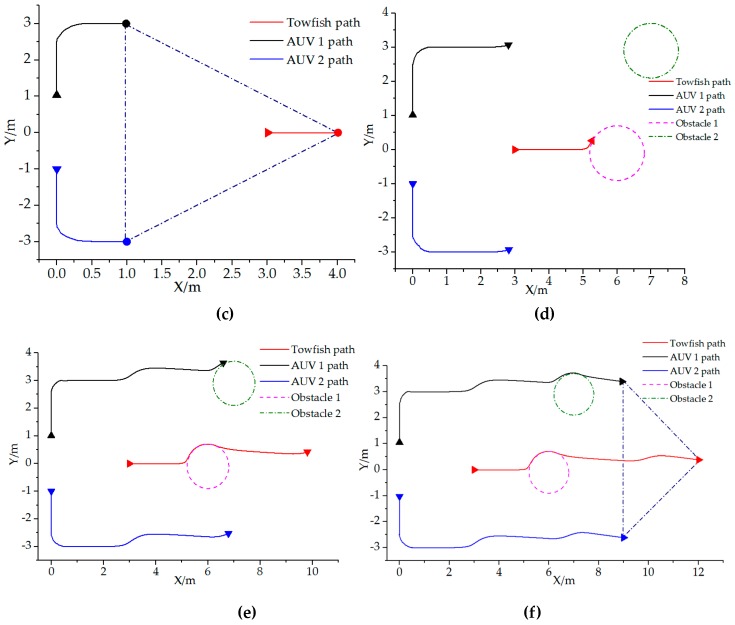
Details of the simulation results: (**a**) Distances between different vehicles; (**b**) Changes of yaw angle of vehicles during the simulations; (**c**) The designed formation is formed when the leader towfish reaches 4 m; (**d**) The formation starts to avoid the first obstacle; (**e**) The formation starts to avoid the second obstacle; (**f**) The formation can be formed again after the two obstacles are avoided.

The initial position of the leader towfish is $x_{1}=\eta_{towfish}={\left[3\;0\;0\right]}^{T}$; AUV 1 is $x_{2}=\eta_{AVU1}={\left[0\;1\;\pi /2\right]}^{T}$ and AUV 2: $x_{3}=\eta_{AVU2}={\left[0\;1\;-\pi /2\right]}^{T}$. Moreover, incidence matrix D of the joint formation vehicles is similar to the above case. The designed triangular structure formation is $X^{f}={\left[\begin{array}{l} \begin{array}{cc} 4 & 0 \end{array}\\ \begin{array}{cc} 1 & 3 \end{array}\\ \begin{array}{cc} 1 & -3 \end{array} \end{array}\right]}^{T}$. Additionally, positions and diameter for the tow obstacles are $Obstacle={\left[\begin{array}{l} \begin{array}{ccc} 6 & 0 & 0.8 \end{array}\\ \begin{array}{ccc} 7 & 3 & 0.8 \end{array} \end{array}\right]}^{T}$. In this case, the final destination is $\eta_{t\arg et}={\left[15,\;0,\;-\right]}^{T}$, where the towfish will lead the other two AUVs after the formation is set. The formation mission velocity is v(t)=1. Results are shown in Figure 12 and Figure 13. The minimum safety distance d is set as 0.5 m.

From Figure 12, we can confirm that the desired formation can be achieved at the startup stage. As the formation moves to the target, the towfish and AUV can avoid the obstacle actively, and the scattered vehicles can be reformed as a joint formation when the two obstacles are avoided. Figure 13a shows the distance changes between the towfish and AUVs, indicating that the distances change during the obstacle avoidance period but can reach the set value after the stage. The leader towfish manifests almost limited changes except for minimal fluctuations during the obstacle avoidance period. However, the two AUVs change gently with fluctuations during the advancement toward the target from Figure 13b. Moreover, Figure 13c reveals that the formation is formed before they start to move to the target. The towfish and AUV can avoid obstacles from Figure 13d,e, and the formation can be reformed successfully from Figure 13f.

## 5. Conclusions

In this study, the triangular structure joint formation that combines a towfish and tow AUVs is designed based on graph theory. This new type of composite formation has advantages of route flexibility, strong endurance, and real-time communication and can reduce or avoid data loss and time delay. Formation obstacle avoidance was accomplished using the NSB method to keep vehicle integrity. Then, the stability of both methods were proven, and that of the total control scheme was analyzed qualitatively. Results show that scattered vehicles can form the desired formation shape, and the towfish and AUV can avoid obstacles actively. Moreover, the formation can be reformed when vehicles reach the final target. The reliability and practicability of the proposed method was confirmed. However, external disturbances and dynamic obstacles were not considered in this study, and further work will be conducted for an in-depth study.

The simulation results tell us that if there is adequate preparation, this theory could be used in large scale scenarios. Our laboratory is preparing for a small real test and a large scale experiment, and further research on this kind of formation is still ongoing.

## Appendix A

In order to read and understand the paper easily, a table of representative symbols is added in this appendix, shown in Table A1.

**Table A1:** Table of symbols.

Symbols	Meaning	Symbols	Meaning
μ	surge	σ	Control variable
v	sway	J(η)	Jacobian matrix
r	yaw	Λ	gain matrix
$A_{k}$	target set	$\sigma_{d}$	desired value
D	Incidence matrix	$v_{d}$	desired velocity

## Appendix B

The definition of passivity is stated in this appendix so that the importance and concept of “passivity” can be understood easily.

1. Definition of passivity of static nonlinearity:

A static nonlinearity y=h(u), where $h:{\mathbb{R}}^{p}\to {\mathbb{R}}^{p}$, is passive if, for all $u\in {\mathbb{R}}^{p}$,

(A1) $$u^{T}y=u^{T}(h)\;\geq 0.$$

and strictly passive if (A1) holds with strict inequality ∀u≠0.

2. Definition of passivity and strict passivity of dynamical systems:

The dynamical system

(A2) $$H:=\left\{ \begin{array}{l} \dot{\xi }=f(\xi ,u)\\ y=h(\xi ,u)\;\;\;\xi \in {\mathbb{R}}^{n},\;u,y\in {\mathbb{R}}^{p} \end{array}\right.$$

and is said to be passive if there exists a $C^{1}$ storage function S(ξ)≥0 such that

(A3) $$\dot{S}=\nabla S{\left(\xi \right)}^{T}f(\xi ,u)\leq -W(\xi )+u^{T}y.$$

for some positive semidefinite function W(ξ). We say that (A2) is strictly passive if W(ξ) is positive definite.
